# Some Medical Difficulties of Life Assurance

**Published:** 1918-02-23

**Authors:** 


### The Hospital

The Workers' Newspaper of Administrative Medicine and Institutional Life, Administration, National Insurance and Health.

No. 1655, Vol. LXIII. SATURDAY, FEBRUARY 23, 1918. PRICE ONE PENNY

### SOME MEDICAL DIFFICULTIES OF LIFE ASSURANCE.

In his presidential address to the Life Assurance Medical Society Dr. F. Parkes Weber alludes to a number of diseased conditions which may readily escape the ordinary examination even when this is conducted with competence and care, and in so doing he displays the difficulties of the art of diagnosis, more particularly when this has to be applied to organic changes in their early beginnings. On such a topic Dr. Weber speaks with very special authority. He has long distinguished himself by great thoroughness of clinical method and by a quite exceptional knowledge of rare and unusual forms of disease. The medical journals and the transactions of various societies bear impressive testimony to this effect, for they enclose many records of curious cases which Dr. Weber has carefully studied and the recognition and identification of which were due to his clinical acumen and knowledge. Moreover, it is particularly worthy of note in these days of "specialism" that Dr. Weber cultivates a wide outlook on the professional field and that he speaks with the note of an expert in more than one department. When an authority with such qualifications describes difficulties in diagnosis we may well listen to what he has to say, for we may be sure that the difficulties really exist and are not due to any failure either in the methods or compass of the examination.

Yet it has to be remembered that in medical examination for purposes of life assurance there are certain hindrances to thoroughness which do not exist when a sick man seeks help from a physician and desires examination in order that his sufferings may be interpreted and relieved. In this latter instance the examinee has no motive for concealment, and it is his desire that all possible means of investigation shall be practised. His interest is to co-operate with the physician to whom he has come of his own choice, and the more time and attention given to his case the more he is pleased. The candidate for life assurance is in a very different position. In the great majority of instances he regards himself as perfectly well, and is inclined to look upon the medical examination as a quite unnecessary annoyance. The more thorough and searching the test the greater is the measure of his resentment. In occasional instances he is aware that something may be discovered which will interfere with his proposal, and he sets himself to mislead the doctor on this point. Then, there is the strictly business side of the transaction. Not a few of the candidates have been shepherded into the medical officer's room as the result of much importunity on the part of those who have a financial interest in the securing of the prey. If in this room the victim has to meet a complete apparatus of modern diagnostic methods he may shrink from the interview, and in any event his recital of the experience will hardly be calculated to induce his friends to follow his example. In a word, the thoroughness and attention which are welcomed by the private patient are resented by the ordinary candidate for life assurance, and this consideration as a matter of business has to be recognised. Assurance companies want clients, and they want healthy clients. But if they make the conditions of entrance too severe or too objectionable they will seriously limit the number of the applicants. They want to effect sound transactions, and sound transactions in the largest possible numbers. Hence their ideal is the maximum of safety consistent with an abundant *clientèle*. Their pathway is therefore a middle one —a medical examination certainly, and even a thorough medical examination, but not one so rigorous as will frighten customers from their doors.

Medical science may say that even in the man who feels himself thoroughly fit, and who, too, looks the part, there may be hidden the beginnings of mischief which will sooner or later reveal itself in some overt and threatening fashion. To be perfectly sure on this point, or even to be approximately sure, an ordinary medical examination is not sufficient. The ophthalmoscope, the laryngoscope, the sphygmometer, the sphygmograph, the polygraph, and possibly other "scopes" and "graphs," must be given an opportunity; a specimen of blood must be scrutinised under the microscope after being duly stained, and the blood serum must be subjected to laboratory investigation; nor may the advisability of puncturing the spinal canal and examining the cerebro-spinal fluid always be avoided; and, still again, there remains the penetrating ability of the *x*-rays, either with or without the auxiliary of the bismuth meal. All these a suffering patient will, on advice, gladly undergo, for they seem to point the way to what he desires—namely, restored health. But set such a formidable apparatus of investigation at the gate of the avenue which leads to a policy of life assurance and it is certain that many candidates will retreat from the prospect. Even to the medical examination as now arranged there is frequent objection and protest. But multiply the investigation to some such extent as is hinted above and it may be questioned whether such a demand would not bring the business of life assurance to an abrupt and ignominious conclusion. What doctors can find out and certify is an element in the business, but so also is what clients will stand; and the directors as shrewd men of the world have to take due count of both factors. If they close their ears to the doctors they run, undoubtedly, some risk of loss; but if they terrify the clients a still more radical misfortune awaits them.

Such are the general considerations which have to be recognised when a plea is made for more thorough methods in medical examination for life assurance. The scientific mind warms to the invitation, but as a business proposition the proposal has to face the question—Will it work? Life assurance, in short, must have within its compass some amount of scientific medicine, but too much would be the death of it.

